# A Novel Bacteriophage Lysin-Human Defensin Fusion Protein Is Effective in Treatment of *Clostridioides difficile* Infection in Mice

**DOI:** 10.3389/fmicb.2018.03234

**Published:** 2019-01-11

**Authors:** Zhong Peng, Shaohui Wang, Mussie Gide, Duolong Zhu, Hiran Malinda Lamabadu Warnakulasuriya Patabendige, Chunhui Li, Jianfeng Cai, Xingmin Sun

**Affiliations:** ^1^Department of Molecular Medicine, Morsani College of Medicine, University of South Florida, Tampa, FL, United States; ^2^State Key Laboratory of Agricultural Microbiology, College of Animal Science and Veterinary Medicine, Huazhong Agricultural University, Wuhan, China; ^3^Department of Chemistry, University of South Florida, Tampa, FL, United States

**Keywords:** *Clostridioides difficile* infection, lysin-human defensin fusion protein, lytic activity, treatment, novel bacteriophage

## Abstract

*Clostridioides difficile* is the leading cause of worldwide antibiotics-associated diarrhea. In this study, we report the construction and evaluation of a novel bacteriophage lysin-human defensin fusion protein targeting *C. difficile*. The fusion protein, designated LHD, is composed of two parts connected by a 3-repeating unit linker “(GGGGS)_3_”: the catalytic domain of a lysin protein from a *C. difficile* bacteriophage phiC2 (LCD), and the functional domain of a human defensin protein HD_5_. Lytic assays showed that LHD protein had a potent lytic activity against different types of clinical *C. difficile* strains, including the epidemic 027, 078, 012, and 087 strains. The minimum inhibitory concentration (MIC) of LHD was 0.78 μg/ml, which was lower than the MIC of the protein LCD (1.56 μg/ml), and the MICs of metronidazole (4 μg/ml) and vancomycin (4 μg/ml). In addition, the LHD protein could lyse *C. different* strains in different pHs (6.0, 7.0, and 8.0). Evaluation of LHD potency *in vivo* using mouse model of *C. difficile* infection (CDI) showed that administration of the LHD protein (twice daily for 7 days) was effective in mitigating the symptoms and reducing the death from CDI. Treatment with LHD also significantly decreased the number of *C. difficile* spores and the toxin level in feces from the infected mice. Our data suggest that this novel lysin-human defensin fusion protein has a potential on CDI control.

## Introduction

*Clostridium difficile*, reclassified as *C. difficile* (Lawson et al., 2016), is a Gram-positive, spore-forming, anaerobic, and toxin-producing nosocomial pathogen. Since the first description of a *C. difficile-*associated disease (CDAD)-like case in 1892 (Finney, 1893), *C. difficile* infection (CDI) has become a high-impact health care–associated infection throughout the world, especially in the developed countries. In the United Sates, *C. difficile* is listed as one of the three most urgent antibiotic resistance threats by the Centers for Disease Control and Prevention (CDC) (Centers for Disease Control and Prevention [CDC], 2013), and CDI is responsible for approximately 453,000 cases of infections and 29,000 deaths every year, with an annual economic burden ranging from $436 million to $3 billion dollars (Napolitano and Edmiston, 2017). In Europe, CDI was associated with considerable short or long term disability, 8382 deaths per year (Cassini et al., 2016), and an annual economic burden of €3 billion euro (Reigadas Ramirez and Bouza, 2018).

Currently, oral antibiotics such as metronidazole, vancomycin, and fidaxomicin is still recommended treatment for CDI (Debast et al., 2014; McDonald et al., 2018). However, *C. difficile* isolates with significantly reduced susceptibility, and even resistance to these recommended antibiotics have been frequently identified and reported (Spigaglia, 2016; Peng et al., 2017a,b). In this regard, development of novel antibiotics and/or alternative treatment strategies for CDI receives increasing attentions nowadays. A previous study showed that a prophage lysin PlyCD and its catalytic domain PlyCD1-174 had good lytic activities against specific *C. difficile* strains (Wang et al., 2015). In addition to phage lysin, human alpha-defensin 5 (HD5) can also effectively lyse hypervirulent *C. difficile* strains (Furci et al., 2015). Here, we report the generation of a novel fusion protein containing bacteriophage lysin and human defensin, which showed potent lytic activity *in vitro*, and was effective in treatment of CDI in mice.

## Materials and Methods

### Bacterial Strains

*Clostridioides difficile* strains R20291 (ribotype 027), M120 (ribotype 078), VPI 10463 (ribotype 087; ATCC 43255), CD630 (ribotype 012), LC693 (ribotype unknown, ST201), and 1377 (ribotype 012) were used in this study. Strains M120 and VPI 10463 were provided by Dr. Joseph A. Sorg from Texas A&M University. Strains R20291 and CD630 were provided by Dr. Abraham L. Sonenshein from Tufts University. Strains 1377 and LC693 were epidemic clinical strains in China (Li et al., 2015; Peng et al., 2017c). Detailed information of these *C. difficile* strains is listed in Table 1.

**Table 1:** Information of *Clostridioides difficile* strains used in this study.

Strain	Ribotype	Sequence type	Toxin profile	Country of isolation
R20291	027	ST1	A+B+CDT+	United Kingdom
M120	078	ST11	A+B+CDT+	Ireland
VPI 10463	087	ST46	A+B+CDT-	Canada
CD630	012	ST54	A+B+CDT-	Switzerland
LC693	Undetermined	ST201	A+B+CDT+	China
1377	012	ST54	A+B+CDT-	China


*CDT (binary toxin of *C. difficile*).*

### Construction and Expression of the Fusion Protein LHD and Catalytic Domain (LCD) of *C. difficile* Phage phiC2

The gene sequence coding for catalytic domain (LCD, 179 aa) of *C. difficile* phage phiC2 (NCBI reference sequence NC_009231), and the fusion gene sequence (LHD) coding for LCD, a 3-repeating unit linker (GGGGS)_3_ and human alpha-defensin 5 (HD5) (32 aa) (Furci et al., 2015) were synthesized, and optimized for expression in *Escherichia coli* by GenScript. The synthesized fusion gene sequence, and gene sequence coding for LCD were cloned into pET-28a (+) using restriction enzymes *Bam*HI and *Hind*III. The recombinant plasmids pET-LHD and pET-LCD were introduced into *E. coli* BL21DE3 for protein expression. The resultant proteins LHD and LCD carry an N-terminal His-tag. For protein induction and expression, *E. coli* BL21DE3 cells carrying pET-LHD or pET-LCD were inoculated into LB broth containing 50 μg/mL kanamycin, and incubated at 37°C with shaking to mid-log phase (OD_600_ values between 0.6 and 0.8), followed by addition of 0.5 mM IPTG to induce protein expression for 2–3 h. To determine the best inducing condition for each of the proteins, bacteria with IPTG were grown at 15°C for overnight; 20°C, overnight; 25°C, overnight; 30°C, 3 h; 30°C, 4 h; 37°C, 3 h, and 37°C for 4 h.

After induction, bacterial culture was centrifuged, and the pellets were re-suspended in a lysis buffer (Buffer A: 0.5 M NaCl+0.02 M Na_3_PO_4_; PH = 7.5). Then, the bacterial suspension was sonicated, and centrifuged at 20,000 rpm for 40 min. The supernatant recovered after centrifugation was filtered through a 0.45-μm membrane, the proteins were purified using a GE Healthcare HisTrapHP^TM^ Nickel column (Uppsala, Sweden) with 5, 10, 15, 30, and 100% of elusion buffer (Buffer B: Buffer A+500 mM imidazole, PH = 7.5). The purified proteins were dialyzed at 4°C against PBS overnight, concentrated using Centricon centrifugal filter (Millipore), and stored in PBS at -80°C for further study. The protein purity was analyzed by SDS-PAGE, and concentration determined by nanodrop.

### Determination of Lytic Activity of Proteins LHD and LCD Against *C. difficile* Strains

The lytic activity of proteins LHD and LCD against *C. difficile* strains was determined as previously described (Wang et al., 2015). Briefly, *C. difficile* strains were cultured in an anaerobic chamber to mid-log phase, and the bacterial pellets were harvested by centrifugation at 3000 × *g* for 5 min. Pellets were washed twice and re-suspended in sterilized ddH_2_O, and resuspensions were not buffered. Prior to tests, bacterial optical density at 600 nm (OD_600_) was adjusted to approximately 0.8–1.0. Proteins LHD and LCD were added into the cell re-suspension with the final concentration of 200 μg/ml. The drop in OD_600_ at 37°C was measured every 10 min for 60–90 min. Bacterial re-suspension with sterilized water was also set as a control. Three repeats were included in each test.

The minimum inhibitory concentrations (MICs) of LHD and LCD were determined using the protocol of Wiegand et al. (2008) with some modifications, as described previously (Gilmer et al., 2017). In brief, strain R20291 was grown in BHIS and adjusted to -5 × 10^5^ cells/ml in BHIS, and distributed into the wells of a 96-well round bottom polystyrene microtiter plate. In the wells of each row, either sterile-filtered lysin or control vehicle (metronidazole, vancomycin, and fidaxomicin) was added with a final concertation varied from 200 to 0.19 μg/ml (2-fold dilution) (Wiegand et al., 2008). The plates were incubated at 37°C for 18 h. The MIC was “the lowest or minimum concentration of lysin or control vehicle that prevented the formation of a cell pellet (a measure of growth) on the bottom of the wells” (Gilmer et al., 2017). The MICs were also confirmed by measuring the OD_600_ values using a Bio-Rad plate reader (Bio-Rad, Hercules, CA, United States).

To determine the optimal pH values for the lytic activity of LHD against *C. difficile*, optical drop assays described above were performed with *C. difficile* strain R20291 in buffers of different pH (pH 6.0, 7.0, and 8.0).

### Determination of Inhibitory Activity of LHD Against *C. difficile* Toxin B (TcdB)

It was reported that the human defensin protein HD5 (HD) could inhibit TcdB (Giesemann et al., 2008). Therefore, we evaluated the inhibitory effects of LHD and HD on TcdB. Briefly, CT26 cells in 12-well plates were exposed to LHD, synthesized HD or PBS at 750 ng/ml (50 times of TcdB concentration) for 1 h, followed by exposure to TcdB at 15 ng/ml for 5 h. Total cell lysates were subjected to 12% SDS-PAGE separation, and transferred onto a Nylon membrane. Following blocking for 1 h at room temperature with 5% skim milk, the membrane was incubated overnight at 4°C with RAC1 antibody (1: 1000, Cat: 610650, and BD Biosciences) and β-actin antibody (1: 10000, Cat: A5441, and Sigma-Aldrich). After washing PBST (PBS with 0.1% Tween), the membrane was incubated with horseradish peroxidase-conjugated secondary antibody goat anti-mouse (Cat: ab97023, goat anti-rabbit, IgG, 1:3000, and Abcam), the antibody-reactive bands were revealed by enhanced chemiluminescence detection on Hyperfilm (Thermo Fisher Scientific, Waltham, MA, United States).

Glucosyltransferase (GT) activity of TcdB was also measured by its ability to glucosylate Rho GTPase Rac1 in cell lysates (Zhang et al., 2013). CT26 cell pellets were resuspended in a reaction buffer (50 mM HEPES, pH 7.5, 100 mM KCl, 1 mM MnCl_2_, and 2 mM MgCl_2_), and lysed by passing through a 30 G needle for 40 times. After centrifugation (167,000 g, 3 min), the supernatant was used as a cytosolic fraction (protein concentration 2.5 mg/ml). To perform the glucosylation assay, the cytosolic fraction was incubated with TcdB at 15 ng/ml (with or without HD at 750 ng/ml or LHD at 1500 ng/ml) at 37°C for 60 min. The reaction was terminated by adding SDS-sample buffer, and samples were heated at 100°C for 5 min before loading on a 12% SDS-PAGE gel. Western bot analysis was performed as described above to detect non-glucosylated Rac1.

### Evaluation of Treatment Efficacy of LHD in the Mouse Model of CDI

C57BL/6 mice (6-week-old) were purchased from Charles River Laboratories, MA, United States. The mice were housed in groups of 5 animals per cage under the same conditions. All studies followed the Guide for the Care and Use of Laboratory Animals of the National Institutes of Health, and were approved by the Institutes Animal Care and Use Committee (IACUC) at University of South Florida under the animal protocol number IS00003756. Mouse model of CDI was established as described previously (Zhang et al., 2015). The experimental design is illustrated in Figure 6. Briefly, twenty BL6/C57 female mice were divided into two groups (*n* = 10). Before challenge, mice were pre-treated with antibiotics mixture [ampicillin (200 mg/kg), kanamycin (40 mg/kg), gentamicin (3.5 mg/kg), colistin (4.2 mg/kg), metronidazole (21.5 mg/kg), and vancomycin (4.5 mg/kg)] in drinking water for 5 days. After that, all mice were given autoclaved water for 2 days, followed by a single dose of clindamycin (10 mg/kg) intraperitoneally 1 day before (day-1) challenge with *C. difficile* R20291 spores at 10^6^ by gavage (day 0). At 4-h post infection, one group of mice (R20291+LHD) were given a dose of 400 μg LHD in 200 μL of PBS by gavage, and another group of mice (R20291+PB) received sterilized PBS as control. From the first day (day 1) to the seventh day (day 7) after infection, mice were given a dose of 400 μg LHD or PBS by gavage twice a day. Weight changes, diarrhea, and survivals of the mice were recorded during the 7-day monitoring period. Fecal samples from mice were collected at days 0, 1, 3, 5, and 7 post challenge for quantitating *C. difficile* spores and TcdA/TcdB concentration.

To appreciate the stability and activity of LHD in the mouse intestine, LCD protein was incuated with freshly prepaerd mouse intestinal contents for 15, 30, and 60 min, and retrived by centrifugation. Lytic activities of LHD treated with intestinal contents were determined on strain *C. difficile* R20291 as described above.

### Quantification of *C. difficile* Spores From Mouse Feces

Fecal samples were collected on days 1, 3, 5, and 7 post-infection. 50 mg of feces were dissolved with 500 μl sterile MilliQ water for 16 h at 4°C, and then treated with 500 μl of purified ethanol (Sigma-Aldrich) for 60 min at room temperature to kill vegetative cells. Samples were vortexed, serially diluted and plated onto selective medium supplemented with taurocholate (0.1% w/v), Cefoxitin (16 μg/mL), L-cycloserine (250 μg/mL). The plates were incubated anaerobically at 37 for 48 h, colonies counted and results expressed as the CFU/gram of feces.

### Quantitation of *C. difficile* Toxins in Mouse Feces

After challenges with *C. difficile* spores, feces were collected and dissolved in an equal volume (g/ml) of sterile PBS containing protease inhibitor cocktail and the supernatants were collected after centrifugation and stored at -80°C. TcdA/TcdB concentrations in the fecal samples of Tcd169 or Tcd169FI-immunized mice were determined by ELISA. Brieflly, 96-well Costar microplates were coated with 100 μl of anti-TcdA antibody (1 μg/ml) and anti-TcdB antibody (1 μg/ml) overnight in phosphate-buffered saline (PBS) at 4°C. On the next day, each well was blocked with 300 μl of blocking buffer (PBS + 5% dry milk) at RT for 2 h. Next, standards and samples were added to each well (100 μl) in duplicate and incubated for 90 min at 25°C. After another set of washes, HRP-chicken anti-*C. difficile* Toxin A or B (1:5,000 dilution in PBS, Gallus Immunotech, Shirley, MA, United States) was added to wells for 30 min at RT. A final set of 3 washes preceded the addition of the TMB Microwell Peroxidase Substrate for 20 min at RT in the dark. The reaction was stopped with 2 N H_2_SO_4_, and the absorbance was measured using a plate reader at 450 nm, and the ELISA was analyzed by a spectrophotometer at 450 nm utilizing BioTek Gen5 Version 2.0 Data Analysis Software.

### Statistical Analysis

When comparing results for two groups, student’s unpaired *t*-test was used for statistical significance; when comparing the results of more than two groups, ordinary one-way analysis of variance (ANOVA) with *post-hoc* analysis by Dunnett’s test was used. Differences were considered statistically significant if *P* < 0.05 (^∗^). All statistical analyses were performed using GraphPad Prism software.

## Results

### Design and Expression of the Lysin-Human Defensin Fusion Protein, LHD

**FIGURE 1:**
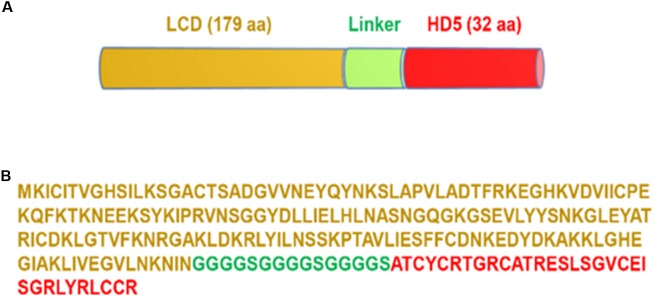
Design of LHD fusion protein. **(A)** Diagram of LHD containing catalytic domain of the lysin from phage phiC2 (LCD), a linker (GGGGS)_3_, and human alpha-defensin protein HD_5_. LHD has 226 amino acids with a PI of 9.10 and MW of 24.4 kDa. **(B)** Amino acid sequence of LHD; sequence of LCD is marked in gold; sequence of the linker is marked in green; and sequence of the HD_5_ protein is marked in red.

The novel bacteriophage lysin-human defensin fusion protein LHD was designed by linking the catalytic domain (LCD, 179aa) of a lysin protein from phage phiC2 (NCBI reference sequence NC_009231) and human alpha-defensin 5 (HD5, designated HD in this paper) (Furci et al., 2015) with a 3-repeating unit linker (“GGGGS”)_3_ (Figure 1). A recent study showed that that phage phiC2 is present in the majority of human isolates of *C. difficile* (Roy Chowdhury et al., 2016), therefore, we used LCD from phage phiC2 to as part of the LHD. There was 34% identity in amino acid sequence between LCD and PlyCD_1-174_. The predicted molecular weight of the lysin-human defensin fusion protein LHD was approximately 24.4 kDa with a PI of 9.1.

The DNA sequences of LHD and LCD were cloned into the protein expression vector pET-28a (+). The recombinant plasmids were transformed into the expression host *E. coli* BL21 cells for protein induction and expression. After induction at 37°C, both LHD and LCD seemed expressed in inclusion bodies, since no LHD or LCD could be detected on SDS-PAGE gels from supernatants recovered from centrifugation of the sonicated bacterial pellets (data not shown).

**FIGURE 2:**
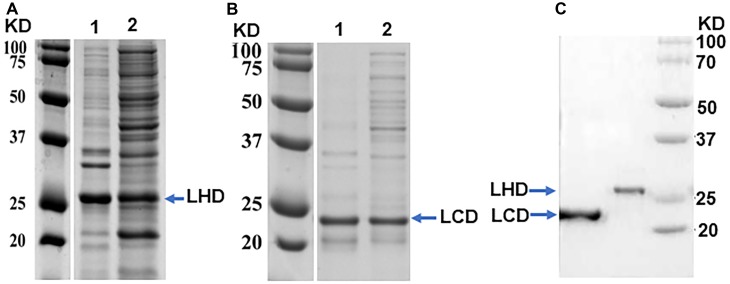
Purification of protein LHD and LCD. **(A)** SDS-PAGE analysis of protein LHD induced and expressed in cell pellet (as inclusion bodies) (Lane 1) and supernatant (soluble form) (Lane 2) at 15°C, overnight. **(B)** SDS-PAGE analysis of protein LCD induced and expressed in cell pellet (as inclusion bodies) (Lane 1) and supernatant (soluble form) (Lane 2) at 37°C for 3 h. **(C)** SDS-PAGE gel analysis of purified LHD and LCD.

After an overnight induction at 15°C, protein LHD was detected in the supernatant after centrifugation of the sonicated bacterial pellets (Figure 2A), indicating some portions of expressed LHD were properly folded. When induced at 30°C for 3 h, protein LCD was well expressed and was also detected in the supernatant after centrifugation of the sonicated bacterial pellets (Figure 2B). Proteins LHD and LCD carry an N-terminal His-tag. After purification of LHD and LCD using GE Healthcare HisTrap^TM^ HP Nickel column, the purity of the proteins was analyzed on SDS-PAGE gels, and the results showed that both purified proteins were ≥95% pure (Figure 2C).

### Lytic Activity of LHD and LCD on *C. difficile* Clinical Strains

**FIGURE 3:**
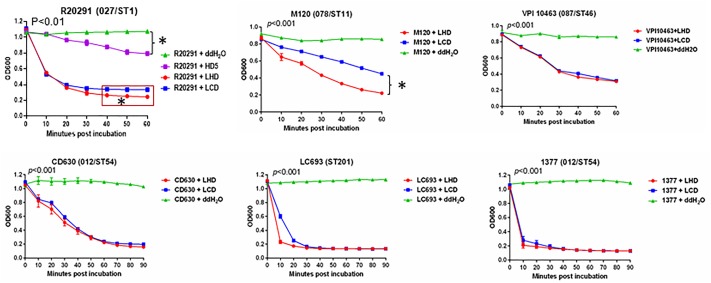
Lytic activity of LHD on different types of *Clostridioides difficile* strains. The lysin catalytic domain LCD was included as a control. In strain R20291, HD5 was also included as a control. Lytic activity of LHD was significantly higher than that of LCD at the time points marked in red boxes (^∗^*p* < 0.05). *p* < 0.001 between groups “*C. difficile* strain+LHD” and “*C. difficile* strain+H_2_O”. Data are present as “Mean ± SD”. Experiments were repeated 3 times, and representative data were shown.

Initially, we determined the lytic activity of LCD and LHD on a *C. difficile* 027 strain, R20291, including HD5 as a control. The optical drop assays showed that both LHD and LCD had potent lytic activities on R20291 at a concentration of 200 μg/ml, with LHD displaying a better lytic activity (Figure 3). While HD5 also showed moderate lytic activity compared to strain R20291 treated with ddH_2_O), it was much less potent than both LHD and LCD in lysing strain R20291 cells (Figure 3). We wondered whether LHD or LCD is also potent against other ST type of epidemic *C. difficile* strains. To this end, we further evaluated lytic activities of LHD/LCD on strains M120 (078/ST11), VPI10463 (087/ST46), CD630 (012/ST54), LC693 (ST201, a novel binary toxin-positive *C. difficile* strain associated with severe diarrhea in China) (Peng et al., 2017c), and 1377 (012/ST54, an epidemic strain in Xiangya hospital in China). Both LHD and LCD proteins showed potent lytic activities on different types of clinical epidemic strains (Figure 3) with LHD being slightly more potent on strains VPI10463, CD630, LC693, and 1377. Interestingly, LHD was more potent than LCD on strain M120 (*p* < 0.05, Figure 3). More impressively, LHD and LCD rapidly and drastically lysed strains LC693 and 1337, two epidemic *C. difficile* strains in China, in 10–20 min (Figure 3).

To test the MIC of LHD protein, optical drop assays were performed using a series of 2-fold-diluted protein LHD (from 200 to 0.19 μg/ml) to lyse a *C. difficile* R20291. The results showed that LHD protein could lyse the bacteria at a concentration as low as 0.78 μg/ml (Table 2). The MIC of the lysin catalytic domain LCD was 1.56 μg/ml, while the MICs of the three treatment antibiotics for CDI including metronidazole, vancomycin, and fidaxomicin were 4, 4, and 0.25 μg/ml, respectively (Table 2).

**Table 2:** Minimum inhibitory concentrations (MIC) of different anti-*C. difficile* agents tested on strain R20291.

Agents	LHD	LCD	Metronidazole	Vancomycin	Fidaxomicin
MIC (μg/ml)	0.78	1.56	4	4	0.25


**FIGURE 4:**
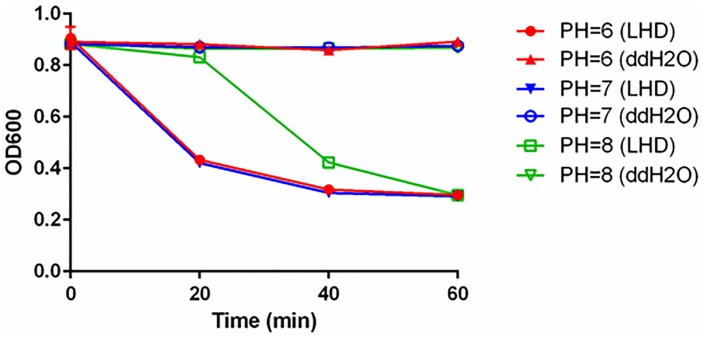
Lytic activity of LHD on *C. difficile* R20291 in different pH conditions. Prior to tests, bacterial optical density at 600 nm (OD_600_) was adjusted to approximately 0.8–1.0. Protein LHD was added into the cell re-suspension with the final concentration of 200 μg/ml. The drop in OD_600_ at 37°C was measured once per 20 min for 60 min. Bacterial re-suspension with sterilized water was also set as a control. Data are present as “Mean ± SD”. Experiments were repeated 3 times, and representative data were shown.

To test the pH sensitivity, the lytic activity of LHD against strain R20291 was measured at different pH conditions. As shown in Figure 4, LHD (200 μg/ml) lysed strain R20291 more efficiently at PHs of 6 and 7. LHD was still partially active at PH of 5, but lost almost all activity at PHs of 2, 3, and 4 (Supplementary Figure S1).

### LHD Inhibits Cytotoxicity of TcdB

**FIGURE 5:**
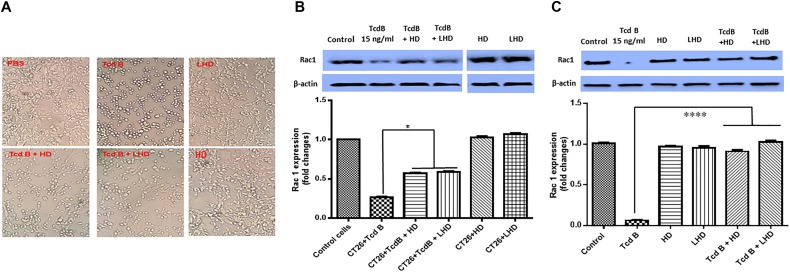
LHD inhibits cytotoxicity of TcdB. **(A)** LHD inhibits TcdB-induced cell rounding. CT26 cells in 12-well plates were exposed to HD or LHD at 750 ng/ml (50 times of TcdB concentration) or nothing for 1 h, followed by exposure to TcdB at 15 ng/ml for 5 h. **(B)** Western-blot analysis of non-glucosylated Rac1 in CT26 cells treated with TcdB in the presence or absence of LHD or HD. TcdB glucosylates Rac1 in cells, serving as a readout for toxin cytotoxicity. Up panel shows one blot being cut into two separate blots to remove extra sample lanes. i.e., all bands were from the same blot. Quantitation of Rac1 levels in Western-blot was shown in low panel (^∗^*p* < 0.05). **(C)** CT26 cells were lysed, and the cytosolic fraction was exposed to TcdB (15 ng/ml) with or without HD at 750 ng/ml (50 times of TcdB concentration) or LHD at 1500 ng/ml (same molecular concentration as HD) or nothing for 1 h followed by Western Blot analysis using a monoclonal antibody that only recognizes non-glucosylated Rac1. β-actin was used as an equal loading control. Quantitation of Rac1 levels in Western-blot was shown in low panel (^∗∗∗∗^*p* < 0.0001).

TcdB has four domains including the N-terminal catalytic glucosyltransferase domain (GT), the autoproteolytic cysteine proteinase domain (CPD), the central translocation domain (TM), and the C-terminal receptor-binding domain (RBD). In the host cells, the CPD domain cleaves GTD off the TcdB, releasing GTD into the cytosol, where GTD glucosylates Rho GTPases including RhoA, CDC42, and Rac1 (Aktories et al., 2017). The CPD-mediated TcdB autocleavage is induced by Inositol hexakisphosphate (InsP6) *in vivo* and *in vitro* (Aktories et al., 2017). It was reported that the human defensin protein HD5 (designated HD in this paper) could inhibit TcdB (Giesemann et al., 2008). We wondered whether LHD carries the anti-TcdB function. To this end, CT26 cells were pre-treated with LHD, HD at 750 ng/ml or PBS as control for 1 h, followed by exposure to Tcd B at 15 ng/ml for 5 h. The results showed that a pre-treatment of LHD or HD decreased cell rounding caused by TcdB (Figure 5A). Western-blot analysis of non-glucosylated Rac1 in cells showed that exposure to TcdB significantly decreased the expression of non-glucosylated Rac1 in cells, as a readout of cytotoxicity of TcdB (Figure 5B). As expected, HD inhibited the cytotoxicity of TcdB. Interestingly, the expression of non-glucosylated Rac1 had a significant increase when the cells received a pre-treatment of LHD prior to exposure to TcdB, indicating that LHD can inhibit cytotoxicity of TcdB as HD does by interfering with GT activity of TcdB.

To exclude the possibility that LHD or HD may interfere with TcdB binding rather than GT activity of TcdB, cytosolic factions of CT26 cells were used as Rac1-containing substrates to confirm that HD or LCD could indeed inhibit GT activity of TcdB. Cytosolic fractions of CT26 cells were exposed to TcdB (15 ng/ml) in the absence or presence of HD (750 ng/ml) or LHD at 1500 ng/ml (same molecular concentration as HD) for 1 h. As shown in Figure 5C, both HD and LHD significantly inhibited GT activity of TcdB to a similar extent, indicating the fused HD portion in LHD is comparable with “free” LHD in inhibiting TcdB glucosylation activity.

### LHD Is Effective in the Treatment of CDI in Mice

**FIGURE 6:**
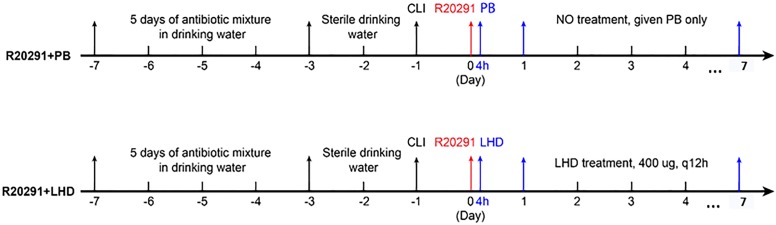
Experimental scheme for evaluation of LHD treatment efficacy in mouse model of CDI. After 5 days of antibiotic pretreatment, mice were given autoclaved water for 2 days, followed by a single dose of clindamycin (10 mg/kg) intraperitoneally, 1 day before (day-1) challenge with *C. difficile* R20291 spores by gavage (day 0). The first group (R20291+PB) was infected with 10^6^ *C. difficile* R20291 spores on day 0, and was given PBS (PB) by gavage at 4 h after spore gavage, followed by administration of PB by gavage twice a day from the first day (day 1) to the seventh day (day 7) after spore gavage. The second group (LHD + R20291) was given 400 μg LHD by gavage at 4 h after spore gavage, followed by treatments of 400 μg LHD in 200 μl of PBS by gavage twice a day from day 1 to day 7 after spore gavage.

To evaluate the treatment efficacy of LHD in mouse model of CDI, 20 of BL6/C57 mice were divided into two groups (*n* = 10). One group was infected with *C. difficile* R20191 spores and treated with LHD, and the other group was infected with R20191 spores and treated with PBS as control. The experimental scheme and treatment plan are illustrated in Figure 6.

**FIGURE 7:**
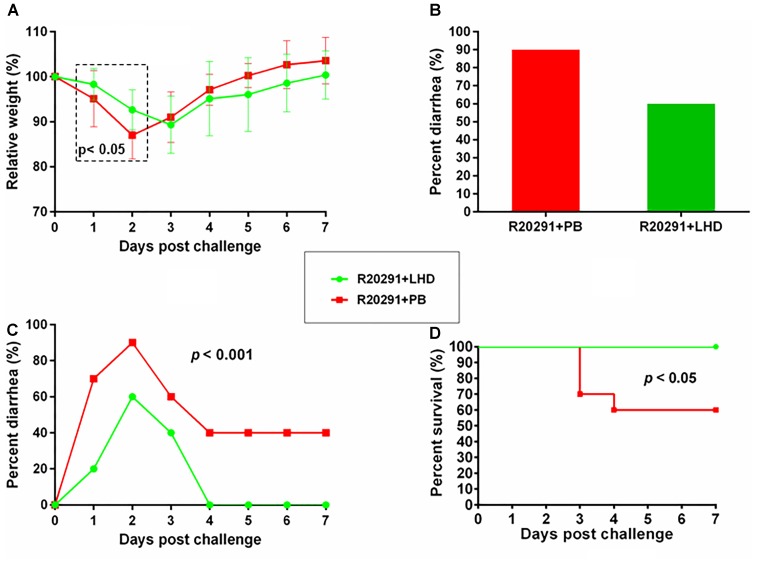
LHD is effective in the treatment of CDI in mice. Twenty BL6/C57 mice were divided into two groups (*n* = 10). One group of mice was infected with *C. difficile* R20291 spores and treated with LHD, and the other group was infected with R20291 spores and treated with PBS as control. The experimental scheme and treatment plan is illustrated in Figure 2, and described in Methods and Materials. Weight changes **(A)**, percentage of diarrhea **(B**,**C)**, survivals of two group mice (**D**) were plotted. Mice in group “R20291+LHD” lost significantly less weight in postinfection days 1 and 2 (*p* < 0.05, marked in dash box region) **(A).**

**FIGURE 8:**
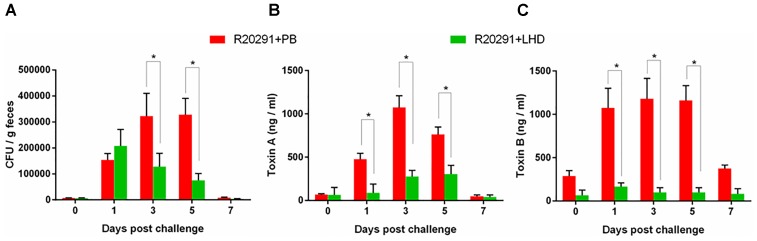
*Clostridioides difficile* spores and toxin levels in fecal samples of mice infected with *C. difficile* R20191 spores with/without LHD treatment. *C. difficile* spore numbers **(A)**, Tcd A level **(B),** and Tcd B level **(C)** in two groups of mice infected with R20291 spores with/without LHD treatment, respectively. Experiments were repeated 3 times, and representative data were shown. Data are present as “Mean ± SD”. ^∗^*p* < 0.05.

**FIGURE 9:**
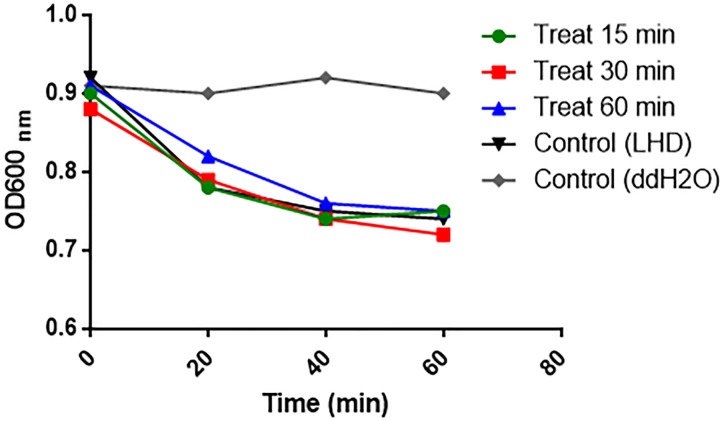
Lytic activity of protein LHD on *C. difficile* R20291 after treatment in mouse intestinal contents. Protein LHD was incubated with mouse intestinal contents at 37°C for 15, 30, or 60 min, then optical drop assays were performed on *C. difficile* R20291 to test the lytic activity of LHD at a concentration of 100 μg/ml. Experiments were repeated 3 times, and representative data were shown.

From the first day of post challenge, weight loss, and diarrhea were observed among mice in both groups (Figures 7A–C). However, treatment with LHD decreased both weight loss and diarrhea rate significantly (Figures 7A,B). The non-treatment group (R20291+PB) showed 90% diarrhea rate, and the diarrhea was observed during the 7-day experimental period (Figures 7B,C). While, 60% of the mice treated with LHD displayed diarrhea, the symptom only lasted 3 days (Figures 7B,C). Death occurred in the non-treatment group (R20291+PB) from the 3rd day of post challenge, and only 60% of the mice finally survived (Figure 7D). However, all mice receiving the treatment of LHD survived during the experimental period (Figure 7D). In addition, treatment with LHD significantly decreased the number of *C. difficile* spores in feces (Figure 8A), and also the toxin-level in feces (Figures 8B,C). To appreciate the stability and activity of LHD in the mouse intestine, LCD protein was incuated with freshly prepaerd mouse intestinal contents for different times, lytic activities of LHD treated with intestinal contents were determined on strain *C.difficile* R20291. As shown in Figure 9, treatment with intestinal contents for 1 h did not affect lytic activity of LHD.

## Discussion

The prevalence and outbreak of CDI have caused serious morbidities and mortalities, and huge economical burdens worldwide (Napolitano and Edmiston, 2017; Reigadas Ramirez and Bouza, 2018). Studies on novel therapeutics such as the use of bacteriophages and their derivatives received increasing attentions. While, phage therapy is proposed to be particularly suited for CDI treatment, the technical difficulties of working with anaerobes limits the research in this area (Hargreaves and Clokie, 2014). A previous study found that both phage lysin protein PlyCD and its catalytic domain PlyCD1-174 displayed good lytic activities on *C. dfficile* (Wang et al., 2015), which sheds light onto the usage of phage derivatives against CDI. In this study, we also found that the catalytic domain of the lysin from phage phiC2 (LCD) and its derivative (LHD) were potent against *C. difficile in vitro* and *in vivo*, indicating the potential of phage lysins as therapeutics in the treatment of CDI.

Although previous study suggested that the PlyCD_1-174_ from *C. difficile* strain CD630 had potential as a novel therapeutic for clinical application against CDI, either alone or in combination with other treatments to improve their efficacy (Wang et al., 2015). We chose the catalytic domain of the lysin from phage phiC2 (LCD) rather than PlyCD1-174 to design the novel bacteriophage lysin-human defensin fusion protein LHD, because phiC2 is present in the majority of human isolates of *C. difficile* (Roy Chowdhury et al., 2016), and it may have a wider lytic spectrum. In fact, both LHD and LCD were potent against different types of *C. difficile* strains, including 027, 078, 087, 012, and ST201 strains (Figure 3). These types of *C. difficile* strains are clinical epidemic strains circulating in different regions of world (Li et al., 2015; Shin et al., 2016). In addition, both LHD and LCD had a lower MIC than the therapy antibiotics metronidazole and vancomycin (Table 2). These findings suggest a good potential of the two proteins present in our study as novel therapeutics against CDI.

In addition to the catalytic domain of the lysin from phage phiC2, another part designed for the lysin-human defensin fusion protein is the functional domain of human alpha-defensin protein HD5 (Figure 1). This region has been documented to inhibit hypervirulent *C. difficile* strains (Furci et al., 2015). Indeed, we found that the lysin-human defensin fusion protein LHD had a lower MIC on *C. difficile* R20291 than the lysin catalytic domain LCD (Table 2).

It has been reported that TcdB is essential for virulence of *C. difficile* (Lyras et al., 2009). A previous study has documented that the human defensin protein HD5 has an inhibitory role on TcdB (Giesemann et al., 2008). Our results showed that the lysin-human defensin fusion protein LHD was comparable to human defensin peptide HD5 in inhibiting cell rounding caused by TcdB (Figure 5A). We further showed that HD and LHD inhibited the GT activity of TcdB to a similar extent (Figures 5B,C).

The potential application of the lysin-human defensin fusion protein in combating CDI was also demonstrated by its treatment efficacy in mouse model of CDI (Figure 7). In this study, we delivered the protein LHD to the *C. difficile*-challenged mice by gavage. Even though the presence of stomach acid might influence the efficacy of LHD on *C. difficile*, however, in the animal experiment, mice were continuously gavaged twice a day for 7 days at 400 μg LHD in 200 μl of PBS per dose. This large volume of PBS may neutralize the stomach acid, protecting LHD there. In addition, as an alkaline protein (*PI* = 9.10), LHD may also neutralize the stomach acid, and stay effective against *C. difficile* in the gut. The weight recovery of the mice receiving LHD was slower than those given PBS at the late stage of the tests (from 3 to 7 dpi, Figure 7A), which might be caused by the administration of LHD (twice a day), limiting the food intake of mice.

In conclusion, we designed a novel lysin-human defensin fusion protein based on the phage lysin protein and human alpha-defensin 5 peptide. Both *in vitro* and *in vivo* tests suggest this novel lysin-human defensin fusion protein has a good potential to help control CDI. To improve the delivery of LHD *in vivo*, we will encapsulate LHD with nanoparticles for our next step of study, we will also evaluate the treatment efficacy of LHD in hamster model of CDI.

## Author Contributions

XS conceived and designed the project. ZP, SW, MG, DZ, HMLWP, and CL performed the experiments. ZP, XS, and SW analyzed the data. ZP, XS, and JC wrote and revised the manuscript. All authors have read and approved the submission of the manuscript.

## Conflict of Interest Statement

The authors declare that the research was conducted in the absence of any commercial or financial relationships that could be construed as a potential conflict of interest.

## Supplementary Material

Figure S1 Lytic activity of LHD on *C. difficile* R20291 in different pH conditions (pH 2–5). Prior to tests, bacterial optical density at 600 nm (OD_600_) was adjusted to approximately 0.8–1.0. Protein LHD was added into the cell re-suspension with the final concentration of 100 μg/ml. The drop in OD_600_ at 37°C was measured once per 20 min for 60 min. Bacterial re-suspension with sterile water was also set as a control. Experiments were repeated 3 times, and representative data were shown.
