# Supplementary figures and images for: A Novel Bacteriophage Lysin-Human Defensin Fusion Protein Is Effective in Treatment of Clostridioides difficile Infection in Mice
Source: Front Microbiol. 2019 Jan 11;9:3234. doi: 10.3389/fmicb.2018.03234 (PMC6336692; doi:10.3389/fmicb.2018.03234)

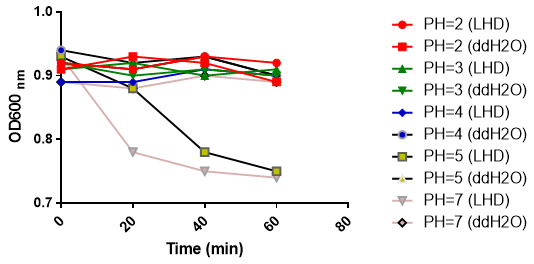

Supplement: Figure S1 — Lytic activity of LHD on C. difficile R20291 in different pH conditions (pH 2–5). Prior to tests, bacterial optical density at 600 nm (OD600) was adjusted to approximately 0.8–1.0. Protein LHD was added into the cell re-suspension with the final concentration of 100 μg/ml. The drop in OD600 at 37°C was measured once per 20 min for 60 min. Bacterial re-suspension with sterile water was also set as a control. Experiments were repeated 3 times, and representative data were shown. [file Image_1.TIF]
